# Music therapy for mental health in children and adolescents: a systematic review and meta-analysis of randomized trials

**DOI:** 10.1186/s13034-026-01102-8

**Published:** 2026-06-23

**Authors:** Zhiyong Zhang, Ming Ye, Lin Zhang

**Affiliations:** 1 Department of Acting, Shanghai Theatre Academy, Shanghai City, China; 2 Shanghai Pulmonary Hospital Tongji University, Shanghai City, China; 3 West China Hospital Sichuan University Jintang Hospital. Jintang First People’s Hospital, No.886 Jinguang road, Jintang County, Chengdu, Sichuan 610400 China

**Keywords:** Child, Adolescent, Music therapy, Depression, Anxiety, Meta-analysis, Randomized controlled trials

## Abstract

**Background:**

Child and adolescent mental disorders impose substantial individual and societal burdens. This study synthesized randomized evidence on the effects of music therapy on depressive and anxiety symptoms, self-esteem, and health-related quality of life in young people, and explored whether intervention characteristics were associated with variations in effect estimates.

**Methods:**

Following PRISMA 2020, this review was retrospectively registered with PROSPERO (CRD420261382585). PubMed, Embase, Cochrane CENTRAL, Web of Science, Google Scholar, ClinicalTrials.gov, and the WHO International Clinical Trials Registry Platform were searched from inception to July 31, 2025, without language limits. Eligible studies were randomized controlled trials enrolling participants aged 6 to 18 years; comparators were no treatment, waitlist, attention control, or usual care; the planned intervention duration was at least four weeks; and outcomes were validated continuous measures at end of treatment. Random-effects models with restricted maximum likelihood generated standardized mean differences (SMD) and 95 percent confidence intervals. Prespecified moderators included age group, session length, session frequency, program duration, and adherence.

**Results:**

Nine trials (n = 690) met inclusion criteria. Music therapy reduced depressive symptoms versus controls (SMD − 0.55; 95% CI, − 0.71 to − 0.38) and improved self-esteem (SMD 0.45; 95% CI, 0.26 to 0.64) and quality of life (SMD 0.69; 95% CI, 0.37 to 1.01). Two trials showed reduced anxiety (SMD − 0.42; 95% CI, − 0.75 to − 0.08). Exploratory subgroup analyses suggested larger effect estimates in younger children and in interventions with longer sessions, higher weekly frequency, longer duration, and higher adherence; however, these findings should be interpreted cautiously given the small number of trials.

**Conclusion:**

Music therapy may offer meaningful benefits for youth mental health, although evidence for anxiety and quality of life is based on a limited number of studies. Findings related to age and intervention delivery characteristics should be considered exploratory and hypothesis-generating rather than evidence of causality or optimal dosing.

**Supplementary Information:**

The online version contains supplementary material available at https://doi.org/10.1186/s13034-026-01102-8.

## Introduction

Mental health during childhood and adolescence is foundational to healthy development, educational attainment, and social participation across the life course. Most adult mental disorders originate by late adolescence, and early-onset conditions are associated with recurrent episodes, poorer academic and occupational outcomes, and elevated risks of self-harm and suicide in young people [1, 2]. Globally, depression and anxiety rank among the leading causes of illness and disability in adolescents, and mental disorders account for a substantial share of disease burden in those aged 10 to 19 years. Recent global burden analyses indicate rising prevalence, incidence, and disability-adjusted life-years attributable to depressive disorders among young people over the past three decades, underscoring the urgency of effective, acceptable, and scalable interventions for this age group [3]. The psychosocial costs extend beyond the individual to families, schools, and communities, with caregiver strain, disrupted learning, and long-term productivity losses. Intervening during childhood and adolescence therefore offers a critical window to modify trajectories, alleviate current symptoms, and reduce the risk of persistence into adulthood.

A range of approaches is recommended for youth depression and related emotional problems, including pharmacotherapy, psychotherapies such as cognitive behavioral therapy and interpersonal therapy, school and community programs, and lifestyle interventions such as structured physical activity [4]. Each approach faces important limitations in real-world settings. Antidepressant medications can produce adverse effects and carry a boxed warning about increased risk of suicidality in children and adolescents, requiring careful monitoring and shared decision making [5]. Psychotherapy, although efficacious, is often constrained by limited service capacity, long wait times, cost, scheduling challenges, and concerns about stigma among young people and caregivers. Even when access is available, engagement and adherence can be difficult to sustain [6]. Exercise-based interventions show promise for symptom reduction but may require extended program duration, frequent sessions, and consistent adherence that are challenging for adolescents to maintain during the school year [7]. Against this backdrop, music therapy has attracted growing interest as a developmentally attuned modality that can be flexibly delivered in schools, clinics, and community settings, elicits high engagement, aligns with adolescent preferences, and is generally safe. Its mechanistic plausibility is supported by evidence that pleasurable music engages reward, motivation, and emotion regulation circuitry, including dopaminergic pathways implicated in anhedonia and affective dysregulation [8].

Controlled trials and reviews have examined music therapy for youth mental health with mixed findings. Several randomized studies report reductions in depressive symptoms and improvements in self-esteem and functioning, whereas others show limited or no benefit, particularly in clinical samples with complex comorbidity [9]. Prior syntheses, including a Cochrane review of music therapy for depression and broader reviews of youth mental health populations, suggest potential efficacy but highlight serious limitations in the evidence base [10]. Common shortcomings include small samples, heterogeneous populations and settings, variability in intervention modalities and intensity, and frequent use of open-label designs. Few reviews have systematically investigated moderators that are highly relevant to implementation, such as age group, session length, frequency, total program duration, adherence, and therapist credentialing. Anxiety, self-esteem, and health-related quality of life are often underreported or pooled inconsistently, and many analyses combine adult and adolescent samples or conflate distinct music-based approaches, obscuring differential effects of active music making versus receptive listening [11]. Methodological issues, including incomplete adjustment for cluster randomization, inadequate reporting to calculate effect sizes, and limited assessment of publication bias, further complicate interpretation [12]. These gaps limit the ability of clinicians, educators, and policy makers to understand how effects may vary across studies and intervention formats.

In light of these limitations, this study aims to provide an updated synthesis of the effects of music therapy on depressive and anxiety symptoms, self-esteem, and health-related quality of life in children and adolescents. The goal is to clarify the magnitude of benefit and to explore whether selected study and intervention characteristics are associated with variation in effect estimates. Any subgroup findings were intended to be exploratory and hypothesis-generating rather than evidence of causality or optimal intervention dosing. This research seeks to inform clinical decisions, guide future trials, and promote equitable access to effective mental health care for young people.

## Methods

This systematic review and pairwise meta-analysis was designed a priori and reported in accordance with the Preferred Reporting Items for Systematic Reviews and Meta-Analyses (PRISMA) 2020 statement [13]. The review was retrospectively registered with PROSPERO (CRD420261382585), with all methods prespecified before data collection and analysis.

### Information sources and search strategy

A comprehensive search was conducted in PubMed, Embase, the Cochrane Central Register of Controlled Trials, Web of Science, and Google Scholar from database inception to 31 July 2025 without language restrictions. To mitigate publication bias, trial registries were additionally screened, including ClinicalTrials.gov and the WHO International Clinical Trials Registry Platform. The search combined controlled vocabulary and free-text terms related to population, intervention, and outcomes. Illustrative terms included child, adolescent, youth, depression, anxiety, mental health, music therapy, rhythm, drum, singing, receptive music listening. The detailed database-specific strategies, search dates, and query logic are provided in Supplementary File 1. Reference lists of eligible reports and relevant reviews were hand-searched to identify additional studies.

All records were imported into EndNote X9 for de-duplication. Titles and abstracts were screened independently by two reviewers. Full-text articles were assessed in duplicate, and disagreements were resolved by consensus or consultation with a third reviewer.

### Study selection

Studies were eligible if they satisfied the following prespecified criteria: (a) Population: children aged 6 to 12 years or adolescents aged 13 to 18 years recruited in any setting, with or without physical or mental comorbidity in order to enhance external validity; (b) Intervention: music therapy delivered by a credentialed music therapist or other trained facilitator, including active modalities such as singing, instrument playing, rhythm based activities, or improvisation, and receptive modalities such as structured music listening, delivered individually or in groups, in schools, clinics, community programs, or laboratory environments, either as a standalone treatment or as an adjunct to usual care provided that the independent effect of music therapy could be isolated. Given the breadth of music-based approaches used in pediatric settings, these interventions were treated as a related therapeutic category for evidence synthesis; (c) Comparator: no treatment, waitlist, attention control, or treatment as usual; (d) Design: randomized controlled trials using individual or cluster randomization with parallel groups; (e) Exposure duration: a minimum planned intervention period of four weeks to estimate sustained rather than acute effects; and (f) Outcomes: at least one validated continuous measure of depressive symptoms, anxiety symptoms, self-esteem, or health related quality of life assessed at end of treatment or at a subsequent follow up.

Studies were excluded if any of the following applied: (a) single session or otherwise acute music exposure protocols intended to capture transient responses; (b) multi component programs that combined music therapy with diet, sleep, physical exercise, mindfulness, or psychotherapy in a manner that precluded attribution of effects to music therapy; (c) nonrandomized designs, quasi experimental studies, crossover trials without an adequate washout, single arm studies, case series, conference abstracts without sufficient data, protocols, reviews, animal experiments, or in vitro studies; or (d) reports that did not provide extractable or derivable summary statistics after author contact. When multiple reports pertained to the same trial, the most complete dataset was used and supplemented with information from companion publications where appropriate.

### Data extraction

After deduplication in EndNote X9, two investigators independently extracted study-level data using a piloted form. Extracted information included bibliographic details, country, setting, trial design, sample size, participant characteristics (age, sex, baseline symptom severity, clinical comorbidity), intervention characteristics (therapeutic modality, session length, frequency, program duration, delivery format, provider credentials), comparator details, adherence, and risk-of-bias domains. For each outcome and time point, means and standard deviations were recorded for intervention and control groups. When studies reported standard errors, confidence intervals, t statistics, interquartile ranges, ranges, or P values, standard deviations were derived using Cochrane-recommended transformations[14]. When only change-from-baseline standard deviations were missing, they were imputed from baseline and post-intervention standard deviations assuming a correlation coefficient of 0.50; sensitivity analyses varied this correlation from 0.30 to 0.70. Authors were contacted up to four times over six weeks for missing or clarifying data.

For multiarm trials, shared comparator groups were split evenly to avoid double counting. For cluster-randomized trials, effect sizes and standard errors were adjusted using the reported intracluster correlation coefficient; if not reported, a conservative value of 0.05 was assumed with sensitivity analyses using 0.01 and 0.10. When multiple instruments assessed the same construct, a predefined hierarchy was applied to select one measure per construct per time point to maintain independence of effects: clinician-rated scales were preferred over self-report, and widely validated pediatric instruments were preferred over less common measures. If both post-intervention and follow-up data were available, the primary analysis used end-of-treatment data; longest follow-up was synthesized in secondary analyses.

### Outcomes

The prespecified primary outcomes were depressive symptoms and anxiety symptoms at the end of the intervention, assessed using validated pediatric instruments. Secondary outcomes included self-esteem and health-related quality of life. Where necessary, scale directions were harmonized so that negative standardized mean differences favored the intervention for symptom outcomes and positive standardized mean differences favored the intervention for self-esteem and quality of life. Because depressive symptoms were measured using different validated instruments, including the BDI/BDI-II, CDI, CES-DC, and DSRS, standardized mean differences were used to place effects on a common metric while preserving the direction of benefit. When multiple time points were available within the end-of-treatment window, the assessment closest to the end of the program was used.

### Risk of bias assessment

The risk of bias in each included RCT was assessed by two independent reviewers using the revised Cochrane Risk of Bias tool for randomized trials (RoB 2) [15]. This tool evaluates potential bias in five domains: (a) the randomization process; (b) deviations from intended interventions; (c) missing outcome data; (d) measurement of the outcome; and (e) selection of the reported result. For each domain, as well as overall, trials were judged as “low risk of bias,” “some concerns,” or “high risk of bias” according to RoB 2 criteria. Disagreements between reviewers were resolved through discussion, with consultation of a third reviewer when necessary.

### Data analysis

All quantitative syntheses were conducted in Stata 17 (StataCorp, College Station, Texas, United States). For continuous outcomes measured on different scales, standardized mean differences with Hedges’ small-sample correction and 95 percent confidence intervals were computed using change-from-baseline scores when available; if not available, post-intervention values were used. Random-effects models were fitted using restricted maximum likelihood to account for between-study heterogeneity. For clinical interpretation, absolute SMDs of approximately 0.20, 0.50, and 0.80 were considered small, moderate, and large effects, respectively. These thresholds serve as descriptive benchmarks rather than outcome-specific minimal clinically important differences, and SMDs may not directly translate to clinically meaningful change across different scales due to differences in content, scoring range, and sensitivity. Statistical heterogeneity was quantified with the between-study variance (tau-squared), the Cochran Q statistic, and I^2^, with I^2^ values of approximately 25 percent, 50 percent, and 75 percent interpreted as low, moderate, and high heterogeneity. For the primary outcomes, 95 percent prediction intervals were additionally calculated to describe the expected range of effects in a new setting.

Predefined subgroup analyses were conducted as exploratory analyses to examine whether effect estimates differed by age group (children versus adolescents), study design (single-blind versus open-label), session length (forty five minutes or less versus more than forty five minutes), session frequency (one session per week versus two sessions per week), program duration (twelve weeks or less versus more than twelve weeks), adherence (at least eighty percent versus less than eighty percent) [16, 17]. Given the limited number of included trials, these analyses were not intended to identify true moderators, establish dose response relationships, or determine optimal intervention parameters.

Small-study effects and publication bias were evaluated visually with contour-enhanced funnel plots and formally with the Egger regression asymmetry test when at least ten studies were available; a two-sided P value less than 0.05 suggested potential small-study effects [18]. Sensitivity analyses were performed using leave-one-out influence diagnostics to examine whether the pooled estimates were unduly driven by any single study. We also conducted a risk-of-bias sensitivity analysis by excluding trials rated as having “some concerns” in the overall RoB 2 assessment; when only one low-risk study remained for an outcome, the result was reported descriptively rather than pooled.

## Results

### Characteristics of included studies

**Fig. 1:**
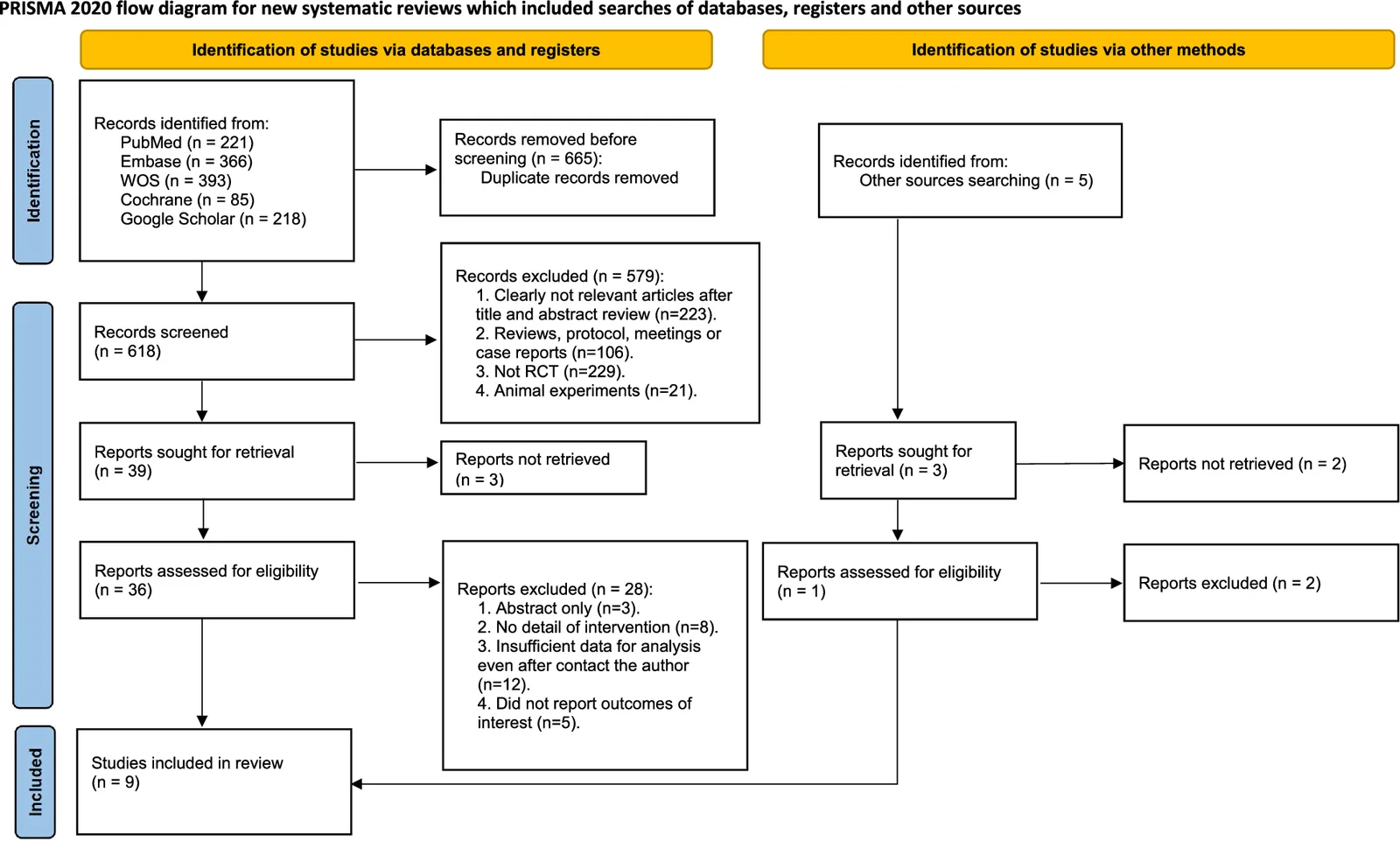
PRISMA flow diagram of study selection

A total of 1,283 records were identified through the initial electronic search. After removing 665 duplicates, 618 articles were screened based on titles and abstracts. Following the exclusion of 579 articles, 39 full-text articles were assessed for eligibility, ultimately leading to the inclusion of 9 RCTs involving 690 children and adolescents in the systematic review and pairwise meta-analysis (Fig. 1) (19—27).

The included studies were published between 2001 and 2024, with a median publication year of 2018. Three studies were single-blind RCTs, and six were open-label RCTs. Sample sizes ranged from 18 to 251 participants, with a median of 60 participants per study. The average age of participants ranged from 9.6 to 13.8 years, with a median of 12.2 years. Four studies included children aged 6–12 years, and five studies included adolescents aged 13–18 years. Intervention durations varied from 8 to 52 weeks, with a median of 12 weeks. The duration of each intervention session ranged from 30 to 90 min, with a median of 55 min. Interventions were delivered once or twice per week, with a median of one session per week. The interventions were heterogeneous and included individual instrumental training, group percussion-based psychotherapy, multimodal music therapy, preference-based music listening programs, free improvisation, and combined active and receptive music therapy approaches. Detailed characteristics of the included studies are provided in Supplementary File 3.

### Results of meta-analysis

#### Depression

**Fig. 2:**
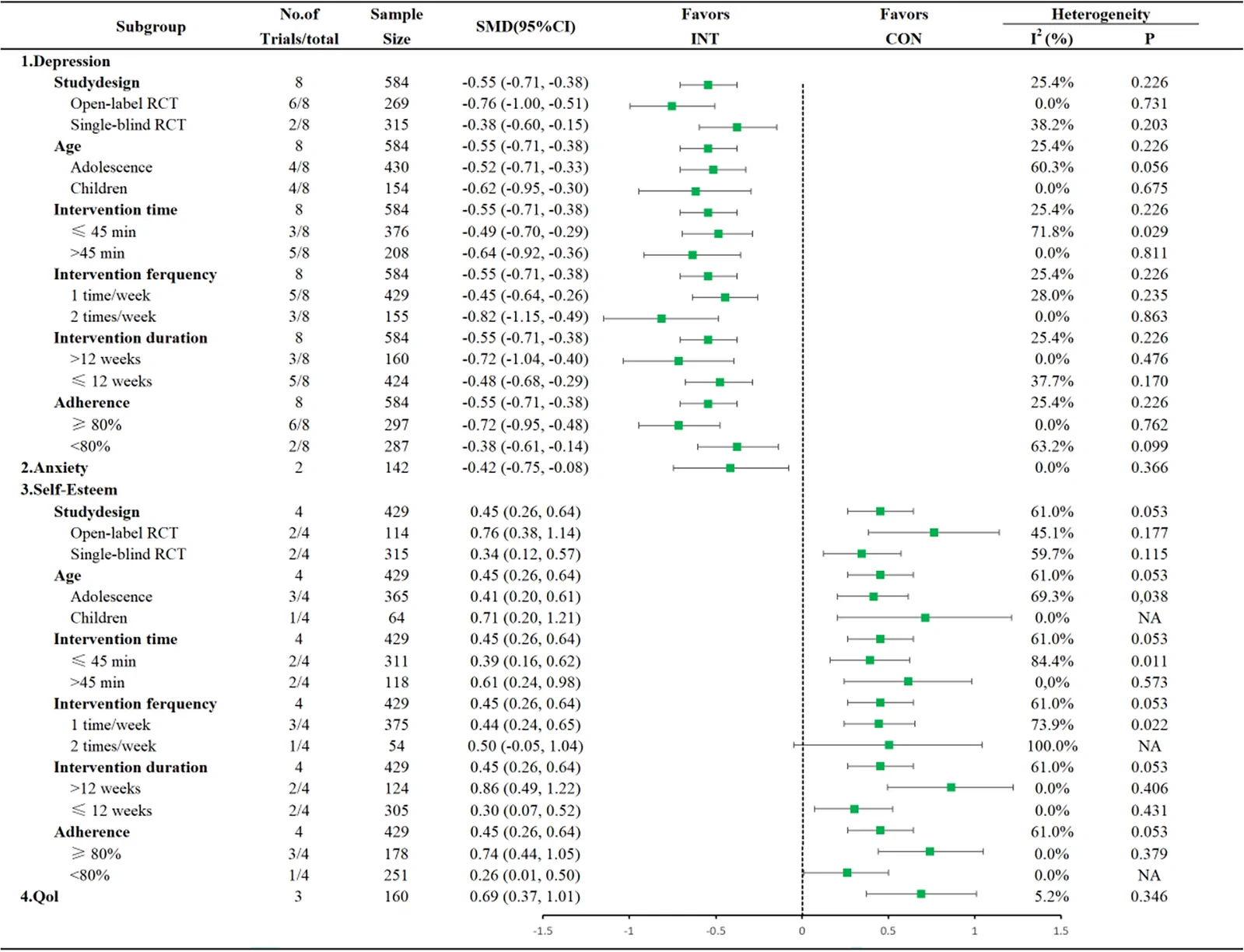
Pairwise meta-analyses of physical activity interventions on postpartum depressive symptoms, anxiety, and quality of life

Eight studies (584 children and adolescents) reported outcomes on the effect of music therapy on depression. The forest plot showed that music therapy significantly reduced depressive symptoms in children and adolescents compared with control conditions (SMD = -0.55, 95% CI -0.71 to -0.38; I^2^ = 25.4%; Fig. 2A). Exploratory subgroup analyses suggested variation in effect estimates across study characteristics; these findings are exploratory, hypothesis-generating, and may be influenced by study-level confounding. Larger effect estimates were observed in open-label RCTs (SMD = -0.76, 95% CI = -1.00, -0.51), among children aged 6–12 years (SMD = -0.62, 95% CI = -0.95, -0.30), with single-session durations greater than 45 min (SMD = -0.64, 95% CI = -0.92, -0.36), with two sessions per week (SMD = -0.82, 95% CI = -1.15, -0.49), for intervention durations longer than 12 weeks (SMD = -0.72, 95% CI = -1.04, -0.40), and with adherence rates ≥ 80% (SMD = -0.72, 95% CI = -0.95, -0.48). These subgroup findings should be interpreted cautiously because several comparisons were based on small numbers of studies. Leave-one-out analysis did not materially change the pooled estimate, suggesting that the depression finding was not driven by any single trial.

#### Anxiety

Two studies (142 children and adolescents) reported outcomes on the effect of music therapy on anxiety. As shown in the forest plot, music therapy significantly reduced anxiety symptoms compared with control conditions (SMD = -0.42, 95% CI -0.75 to -0.08; I^2^ = 0%; Fig. 2B). Due to the limited number of studies, no subgroup analysis was performed for anxiety. These findings are preliminary and should be interpreted cautiously. Leave-one-out analysis indicated limited stability of this finding, likely because only two trials were available.

#### Self-esteem

Four studies (429 children and adolescents) reported outcomes on the effect of music therapy on self-esteem. The forest plot indicated that music therapy significantly improved self-esteem in children and adolescents compared with control conditions (SMD = 0.45, 95% CI 0.26 to 0.64; I^2^ = 61.0%; Fig. 2C), although the moderate heterogeneity indicates some inconsistency across studies. Exploratory subgroup analyses suggested variation by intervention frequency, study design, age, session duration, program length, and adherence; these findings are hypothesis-generating and may be influenced by study-level confounding. Specifically, one session per week significantly improved self-esteem (SMD = 0.44, 95% CI = 0.24, 0.65), while two sessions per week did not show a significant effect (SMD = 0.50, 95% CI = -0.05, 1.04). Other exploratory subgroup comparisons also suggested possible variation across study design, age, intervention time, intervention duration, and adherence. Specifically, larger effects were observed in open-label RCTs (SMD = 0.76, 95% CI = 0.26, 0.64), in children aged 6–12 years (SMD = 0.71, 95% CI = 0.20, 1.21), with single-session durations greater than 45 min (SMD = 0.61, 95% CI = 0.24, 0.98), with intervention durations longer than 12 weeks (SMD = 0.86, 95% CI = 0.49, 1.22), and with adherence rates ≥ 80% (SMD = 0.74, 95% CI = 0.44, 1.05). These findings should be interpreted cautiously given the limited number of included studies. The pooled result remained directionally consistent in leave-one-out analysis, indicating that no single study materially accounted for the overall finding.

#### Quality of life (QoL)

Three studies (160 children and adolescents) reported outcomes on the effect of music therapy on QoL. As shown in the forest plot, music therapy significantly improved quality of life compared with control conditions (SMD = 0.69, 95% CI 0.37 to 1.01; I^2^ = 5.2%; Fig. 2D). Due to the limited number of studies, no subgroup analysis was performed for QoL. These findings should be interpreted cautiously and considered exploratory. Leave-one-out analysis likewise showed no material change in the pooled estimate.

### Risk of bias and publication bias

Among the 9 included RCTs, five studies were assessed as having a low risk of bias overall, while four studies raised some concerns. Regarding the randomization process, eight trials were judged to have low risk, and one trial raised some concerns. In terms of deviations from the intended interventions, five trials had low risk, and four raised some concerns. Seven trials had low risk of bias in missing outcome data, while two raised concerns. For outcome measurement, all nine trials had low risk of bias, and for the selection of reported results, all nine trials had low risk of bias (Supplementary File 4).

Potential publication bias was assessed using funnel plots for depressive symptoms, anxiety symptoms, self-esteem, and quality of life (Supplementary Figures S5.1-S5.4). Visual inspection suggested some asymmetry, particularly for outcomes with fewer studies. Although Egger’s tests were not statistically significant where applicable, the small number of included studies limited the power of these analyses, and small-study effects could not be fully excluded.

### Sensitivity analyses

Leave-one-out analyses showed that the pooled estimates for depressive symptoms, self-esteem, and quality of life were not materially changed by omitting any single study, whereas the anxiety result was less stable because only two trials were available. In the additional risk-of-bias sensitivity analysis, excluding studies rated as having “some concerns” attenuated but did not eliminate the effect on depressive symptoms (4 studies; n = 341; SMD = -0.39, 95% CI -0.62 to -0.15; I^2^ = 0%). The anxiety estimate was unchanged, as both contributing trials were rated as low risk of bias (2 studies; n = 142; SMD = -0.42, 95% CI -0.75 to -0.08; I^2^ = 0%). For self-esteem and quality of life, only one low-risk study remained; these findings were directionally consistent but should be interpreted cautiously (Supplementary Figure S6 and Supplementary Table S6.1).

## Discussion

This systematic review and meta-analysis included nine randomized controlled trials encompassing 690 children and adolescents, providing a comprehensive evaluation of the effects of music therapy on mental health and quality of life in youth. Three principal findings emerged. First, across eight trials, music therapy significantly reduced depressive symptoms relative to control conditions, with a moderate pooled effect. Exploratory subgroup analyses suggested larger effect estimates in open-label designs, among younger children aged 6 to 12 years, when individual sessions exceeded 45 min, when delivery reached two sessions per week, when programs extended beyond 12 weeks, and when adherence was at least 80 percent; these findings are exploratory, hypothesis-generating, and may be influenced by study-level confounding. Second, although only two trials were available, music therapy was associated with a significant reduction in anxiety symptoms, indicating potential transdiagnostic benefits while underscoring the need for additional high-quality evidence in this domain. Third, music therapy yielded meaningful improvements in self-esteem and health-related quality of life. Four trials demonstrated a moderate increase in self-esteem, with exploratory analyses suggesting variation across intervention frequency, session length, program duration, and adherence; these findings are hypothesis-generating and should be interpreted cautiously. Three trials showed significant improvements in quality of life. However, confidence in some estimates is reduced by heterogeneity and variation in outcome instruments. For depressive symptoms, studies used different validated scales, including the BDI/BDI-II, CDI, CES-DC, and DSRS. Although SMDs allowed statistical pooling across these instruments, the scales differ in age targeting, symptom emphasis, scoring structure, and sensitivity to change. Therefore, SMDs may not directly correspond to clinically meaningful change, and caution is warranted when interpreting the magnitude of effects across different scales. Taken together, these findings indicate that music therapy may alleviate negative affective states and support positive psychological functioning and overall well-being in young people. However, because SMDs were calculated across different scales with varying content and sensitivity, the magnitude of effect may not directly reflect clinically meaningful change, and interpretation of effect sizes and delivery-related differences requires caution. Clinically, the pooled effects corresponded to a moderate reduction in depressive symptoms (SMD = -0.55), small-to-moderate improvements in anxiety symptoms and self-esteem, and a moderate-to-large improvement in quality of life. These magnitudes are broadly comparable with those reported for youth psychotherapy and exercise-based interventions, although such comparisons are indirect because of differences in populations, comparators, and outcome measures. Thus, music therapy should be viewed as a promising adjunctive or alternative psychosocial intervention, rather than a replacement for established treatments.

Music therapy offers broad and complementary benefits that span affective regulation, cognitive control, social connectedness, and physiological stress recovery. It is intrinsically rewarding, acceptable to young people, and feasible for delivery in schools, clinics, and community programs, which reduces barriers related to access and stigma while fostering engagement [8]. These attributes are particularly relevant in childhood and adolescence, when developmental plasticity, peer affiliation, and identity formation shape mood trajectories and long-term outcomes [28]. Within this context, the present synthesis indicates that music therapy confers clinically meaningful relief of depressive symptoms in children and adolescents compared with control conditions. Relative to earlier reviews that often mixed adult and youth samples or combined distinct music based approaches, the current evidence focused on young people and examined delivery characteristics that matter for implementation [29]. The convergence of findings with prior randomized studies supports a potentially beneficial signal, while differences in magnitude across reports likely reflect variation in modality, dose, fidelity, and risk of bias [30]. Importantly, the interventions pooled in this review were not uniform, and the summary estimates should therefore be understood as applying to a heterogeneous group of music therapy approaches rather than to any single intervention model. Differences in control conditions across trials, including waitlist, usual care, and attention control, may also have influenced pooled effect sizes and contribute to heterogeneity in observed outcomes. The included interventions also differed in therapeutic modality and delivery format. Active music therapy involves direct musical engagement, such as singing, instrumental playing, rhythm-based activities, or improvisation, whereas receptive music therapy primarily involves structured listening, relaxation, or reflection on music. Similarly, individual sessions may allow closer tailoring to the child’s emotional needs and therapeutic relationship, while group formats may additionally enhance peer interaction, synchrony, belonging, and social support. Because several trials combined active and receptive elements and the number of studies was small, formal comparison by modality or format was not feasible. Therefore, the pooled estimates may mask meaningful differences between active and receptive approaches, as well as between individual and group delivery.

Several mechanisms plausibly underlie the antidepressant effect. Pleasurable music engages reward and salience circuits implicated in anhedonia and recruits frontolimbic networks involved in emotion regulation; repeated practice may strengthen regulatory pathways through experience dependent plasticity [31]. Group music making promotes social bonding and perceived belonging, which buffer depressed affect. Active engagement provides behavioral activation, mastery experiences, and self-efficacy, while rhythmic entrainment supports attentional control and may reduce ruminative processing [32]. Music based activities can also increase parasympathetic activity and attenuate hypothalamic pituitary adrenal axis reactivity, facilitating stress recovery [33]. In addition, music therapy is delivered within a therapeutic relationship in which musical experiences are intentionally shaped, contained, and reflected. This relational context may support emotional expression, strengthen trust and psychological safety, and promote co-regulation, particularly in children and adolescents who may have difficulty communicating distress directly [27]. Together, these pathways offer a coherent account for the improvements in depressive symptoms observed in youth, with benefits likely arising from both the properties of music itself and the therapeutic relationship through which the intervention is delivered. Given that six of nine included trials were open-label, the magnitude of observed effects may be further inflated by expectancy and performance bias, particularly for depressive outcomes, warranting cautious interpretation.

Given the diversity of study designs, participant ages, baseline characteristics, and delivery parameters, prespecified subgroup analyses were undertaken to explore variation in effects. This variation should also be interpreted in light of substantial heterogeneity in the interventions themselves, including differences in modality, format, and therapeutic emphasis across studies. These analyses indicated larger benefits among younger children aged 6 to 12 years, in open label designs, with individual sessions longer than 45 min, with two sessions per week, with total program duration greater than 12 weeks, and when adherence was at least 80 percent. Several considerations may explain these patterns. Younger children may show greater responsiveness because regulatory systems are more malleable, play based learning aligns closely with developmental needs, and caregiver or teacher support can reinforce skills between sessions [34]. Longer sessions likely allow a full therapeutic arc that includes warm up, active music making, reflective processing, and consolidation, which together deepen emotional engagement and skills practice. Higher session frequency may increase exposure to the intervention, but these results do not establish a dose response relationship. Programs extending beyond 12 weeks provide sufficient time for neural and behavioral adaptation, the formation of cohesive group dynamics, and transfer of skills to daily contexts [35, 36]. High adherence is a proxy for both fidelity and total dose; greater participation increases opportunities for mastery, social connection, and stress regulation [37]. Larger effects in open-label trials may partly reflect expectancy or performance bias and should not be interpreted as evidence of greater efficacy. Accordingly, these subgroup findings should be considered exploratory and hypothesis-generating only. They do not establish causality or define optimal intervention parameters.

Anxiety in childhood and adolescence independently undermines learning, peer relationships, and later psychiatric outcomes, making it a priority alongside depressive symptoms. In this meta-analysis, music therapy was associated with a significant reduction in anxiety relative to control conditions across two randomized trials involving 142 participants. Although the number of studies was small, the direction and precision of the effect are consistent with prior youth-focused work showing small to moderate anxiolytic benefits of music-based approaches, while differences in magnitude across reports likely reflect variation in modality, therapist credentialing, and delivery context [38]. Several features of music therapy plausibly account for these gains. Predictable rhythmic structure and contour can reduce uncertainty and promote a sense of safety. Tempo-synchronized breathing and rhythmic entrainment tend to increase vagal tone and reduce sympathetic arousal, which aligns with reductions in somatic anxiety. Auditory attentional capture may interrupt worry cycles and threat-biased processing, and structured listening with guided imagery can rehearse cognitive reappraisal in a low-stakes setting. In active formats, graded performance demands provide gentle exposure to interoceptive cues paired with mastery, while group synchrony affords social buffering that enhances perceived safety [39, 40]. The therapeutic relationship may further support these processes by providing attunement, validation, and emotional containment, helping children and adolescents engage with musical experiences in a more regulated and meaningful way [41]. These attributes offer a coherent explanation for the observed anxiolytic effect and suggest directions for future trials, including clearer specification of musical parameters, incorporation of autonomic and endocrine readouts, and stratification by anxiety phenotype in school and clinical settings.

Self-esteem and health-related quality of life are central to adaptive development in childhood and adolescence, shaping academic motivation, social participation, and resilience in the face of stress. In this review, music therapy was associated with meaningful gains in both domains, indicating benefits that extend beyond symptom relief to the promotion of positive psychological functioning and daily well-being. Compared with earlier reviews that often pooled mixed-age samples or merged diverse arts activities, the present youth-focused synthesis suggests more consistent quality-of-life improvements and a moderate enhancement of self-esteem. The literature has reported similar trends in school-based arts programs and pediatric chronic-illness contexts, although estimates have varied with delivery format and dose; our findings add that improvements are attainable in routine educational and clinical settings and appear robust across diverse instruments for quality of life [42, 43]. Active music making affords repeated mastery experiences, goal setting, and performance accomplishments that cultivate perceived competence and self-efficacy, while collaborative ensemble work strengthens relatedness and social identity, all of which are core drivers of self-esteem within self-determination and social learning frameworks [44]. Opportunities for creative expression and audience feedback may consolidate a positive self-concept and counteract internalized failure narratives common in youth with emotional difficulties. These gains may also be supported by the therapist’s attunement, encouragement, and reflective guidance, which can help children and adolescents feel understood and better able to translate musical experiences into meaningful psychological change. This therapeutic dimension may distinguish music therapy from music engagement without clinical guidance, where relational support and structured reflection may be more limited [45]. The low heterogeneity observed for quality of life is compatible with broad gains across emotional, social, and school-functioning domains when engagement is high and participation is sustained [46]. By contrast, variability in self-esteem effects aligns with subgroup signals: longer sessions and programs allow time to acquire skills and receive credible feedback; adherence amplifies exposure to success experiences; greater effects in younger participants likely reflect higher developmental plasticity and classroom structures that reinforce peer bonding; and the apparent advantage of once-weekly delivery may reflect spacing that preserves novelty, facilitates reflection between sessions, and reduces performance burden, whereas twice-weekly schedules may face adherence constraints or smaller samples in the underlying trials [8]. These observations should be interpreted cautiously, as the subgroup analyses were based on a limited number of trials and were not designed to establish optimal delivery formats. In addition, most studies assessed only end-of-treatment outcomes, with limited or no long-term follow-up, so the durability of improvements in depressive symptoms, self-esteem, and quality of life remains uncertain. This limits conclusions about sustained benefits and underscores the need for future trials to include extended follow-up assessments.

This review offers several strengths that enhance confidence in its conclusions. It followed PRISMA 2020 with prespecified methods, comprehensive searches across multiple databases and trial registries without language restrictions, duplicate screening and data extraction, RoB 2 appraisal, and transparent reporting of search strategies and study flow. It also focused exclusively on children and adolescents and harmonized clinically relevant outcomes across depressive symptoms, anxiety, self-esteem, and health-related quality of life, while prespecifying practice-oriented moderators such as age, session length and frequency, program duration, and adherence, which improves interpretability for schools, community programs, and pediatric services. These strengths notwithstanding, several limitations warrant consideration. The evidence base was uneven across outcomes and designs, with only two trials informing anxiety and three informing quality of life. Six of nine trials were open-label, which may have inflated observed effect sizes through expectancy and performance bias, particularly for depressive symptoms. Small-study effects were difficult to assess due to the limited number of trials, and funnel plot asymmetry suggests that small-study effects cannot be fully excluded despite nonsignificant Egger tests. The additional risk-of-bias sensitivity analysis partly addressed this concern: the depression effect remained significant after excluding studies with “some concerns,” whereas evidence for self-esteem and quality of life became sparse and therefore requires more cautious interpretation. Variation in outcome instruments required synthesis using standardized mean differences. Although this approach enabled pooling across scales such as the BDI/BDI-II, CDI, CES-DC, and DSRS, it could not fully account for differences in scale content, developmental suitability, scoring range, or responsiveness. Together with moderate-to-high heterogeneity for self-esteem and incomplete reporting of variance data or intracluster correlation coefficients in several trials, this leaves residual imprecision; reliance on aggregate data also limited exploration of individual-level moderators. In addition, subgroup analyses were constrained by the small number of included trials and limited precision within several categories, reducing confidence in apparent differences across age and intervention characteristics. Differences in modality and delivery format should also be considered. Active and receptive components, as well as individual and group formats, were not consistently separated across trials, and group size was often insufficiently reported. As a result, session length and total exposure may not have represented comparable therapeutic intensity across studies. Generalizability and durability remain uncertain because most trials prioritized end-of-treatment assessments with limited follow-up, precluding firm conclusions about the persistence of benefit over time, study settings were concentrated in schools and specialty services within a narrow range of countries, reporting on therapist credentialing and adherence supports was variable, and the included music therapy interventions were conceptually and procedurally heterogeneous. This heterogeneity limits attribution to specific therapeutic components and cautions against overgeneralizing the pooled estimates to all forms of music therapy.

## Conclusion

In this meta-analysis of nine randomized trials including 690 young people, music therapy was associated with improvements in depressive symptoms, self-esteem, and health related quality of life, with preliminary evidence of reduced anxiety. Exploratory analyses suggested larger effect estimates in younger children and in interventions with longer sessions, higher frequency, longer duration, and better adherence; these findings are hypothesis-generating and may be influenced by study-level confounding. Evidence for anxiety and long-term durability remains limited, as most trials included only end-of-treatment assessments. The pooled effect for depressive symptoms may have been overestimated, particularly because the predominance of open-label trials could have inflated effect sizes through expectancy and performance bias. Future trials should standardize intervention components, report fidelity and adherence more clearly, extend follow-up, and test potential moderators in adequately powered and, where feasible, blinded studies. Additionally, harmonizing or clearly describing control conditions would improve interpretability, as variation in comparators may influence pooled estimates.

## Supplementary Information

Additional file1 (DOCX 706 KB)

## Data Availability

Data are available on reasonable request.
