# Supplementary material for: Music therapy for mental health in children and adolescents: a systematic review and meta-analysis of randomized trials
Source: Child Adolesc Psychiatry Ment Health. 2026 Jun 23;20:128. doi: 10.1186/s13034-026-01102-8 (PMC13584578; doi:10.1186/s13034-026-01102-8)
Supplement: Supplementary file 1 [file 13034_2026_1102_MOESM1_ESM.docx]

**Supplementary**

Table of Contents:

[Supplementary 1: PRISMA Checklist	1](#_Toc534204563)

[Supplementary 2: Search Strategy	4](#_Toc1894445881)

[Supplementary 3: Characteristics of studies and subjects included in the review	6](#_Toc1347370016)

[Supplementary 4: Risk of Bias	13](#_Toc1262935083)

[Supplementary 5: Publication bias](#_Toc431254745)	14

[Supplementary 6: Sensitive analysis](#_Toc431254745)	15

**Supplementary 1: PRISMA Checklist**

| Item # | Checklist Item | Reported on Page # |
| --- | --- | --- |
|  |  | 1 |
| 1 | Identify the report as a systematic review incorporating a meta-analysis (or related form of meta-analysis). | 1 |
| 2 | Provide a structured summary including, as applicable:  **Background:** main objectives  **Methods:** data sources; study eligibility criteria, participants, and interventions; study appraisal; and *synthesis methods, such as meta-analysis.*  **Results:** number of studies and participants identified; summary estimates with corresponding confidence/credible intervals; treatment rankings may also be discussed. Authors may choose to summarize pairwise comparisons against a chosen treatment included in their analyses for brevity.  **Discussion/Conclusions:** limitations; conclusions and implications of findings.  **Other:** primary source of funding; systematic review registration number with registry name. | 1-2 |
| 3 | Describe the rationale for the review in the context of what is already known*, including mention of why a meta-analysis of randomized controlled trials and dose-response analysis has been conducted.* | 3-5 |
| 4 | Provide an explicit statement of questions being addressed, with reference to participants, interventions, comparisons, outcomes, and study design (PICOS). | 5-6 |
| 5 | Indicate whether a review protocol exists and if and where it can be accessed (e.g., Web address); and, if available, provide registration information, including registration number. | 5 |
| 6 | Specify study characteristics (e.g., PICOS, length of follow-up) and report characteristics (e.g., years considered, language, publication status) used as criteria for eligibility, giving rationale. *Clearly describe eligible treatments included in the treatment , and note whether any have been clustered or merged into the same node (with justification).* | 28-29 |
| 7 | Describe all information sources (e.g., databases with dates of coverage, contact with study authors to identify additional studies) in the search and date last searched. | 5 |
| 8 | Present full electronic search strategy for at least one database, including any limits used, such that it could be repeated. | Supplementary 2 |
| 9 | State the process for selecting studies (i.e., screening, eligibility, included in systematic review, and, if applicable, included in the meta-analysis). | 5-6 |
| 10 | Describe method of data extraction from reports (e.g., piloted forms, independently, in duplicate) and any processes for obtaining and confirming data from investigators. | 6-7 |
| 11 | List and define all variables for which data were sought (e.g., PICOS, funding sources) and any assumptions and simplifications made. | 7-8 |
| 12 | Describe methods used for assessing risk of bias of individual studies (including specification of whether this was done at the study or outcome level), and how this information is to be used in any data synthesis. | 8, Supplementary 4 |
| 13 | State the principal summary measures (e.g., risk ratio, difference in means). Also describe the use of additional summary measures assessed, such as treatment rankings and surface under the cumulative ranking curve (SUCRA) values, as well as modified approaches used to present summary findings from meta-analyses. | 8-10 |
| 14 | Describe the methods of handling data and combining results of studies for each meta-analysis. This should include, but not be limited to:   - Handling of multi-arm trials; - Selection of variance structure; - Selection of prior distributions in Bayesian analyses; and - Assessment of model fit. | 8-10 |
| 15 | Specify any assessment of risk of bias that may affect the cumulative evidence (e.g., publication bias, selective reporting within studies). | 9,  Supplementary 6 |
| 16 | Describe methods of additional analyses if done, indicating which were pre-specified. This may include, but not be limited to, the following:   - Sensitivity or subgroup analyses; - Meta-regression analyses; - Alternative formulations of the treatment ; and - Use of alternative prior distributions for Bayesian analyses (if applicable). | 8-10 |
| 17 | Give numbers of studies screened, assessed for eligibility, and included in the review, with reasons for exclusions at each stage, ideally with a flow diagram. | Figure 1 |
| 18 | For each study, present characteristics for which data were extracted (e.g., study size, PICOS, follow-up period) and provide the citations. | Supplementary 3 |
| 19 | Present data on risk of bias of each study and, if available, any outcome level assessment. | 11,  Supplementary 4, and Supplementary 6 |
| 20 | For all outcomes considered (benefits or harms), present, for each study: 1) simple summary data for each intervention group, and 2) effect estimates and confidence intervals. | Supplementary 3 |
| 21 | Present results of each meta-analysis done, including confidence/credible intervals. Authors may focus on comparisons versus a particular comparator (e.g. placebo or standard care), with full findings presented in an appendix. League tables and forest plots may be considered to summarize pairwise comparisons. If additional summary measures were explored (such as treatment rankings), these should also be presented. | Figure 2, Figure 3, and Figure 4 |
| 22 | Present results of any assessment of risk of bias across studies for the evidence base being studied. | 11,  Supplementary 4, and Supplementary 6 |
| 23 | Give results of additional analyses, if done (e.g., sensitivity or subgroup analyses, meta-regression analyses. | Figure 4,  and Figure 5 |
| 24 | Summarize the main findings, including the strength of evidence for each main outcome; consider their relevance to key groups (e.g., healthcare providers, users, and policy-makers). | 14-19 |
| 25 | Discuss limitations at study and outcome level (e.g., risk of bias), and at review level (e.g., incomplete retrieval of identified research, reporting bias). | 19 |
| 26 | Provide a general interpretation of the results in the context of other evidence, and implications for future research. | 19 |
| 27 | Describe sources of funding for the systematic review and other support (e.g., supply of data); role of funders for the systematic review. | 19 |

PICOS = population, intervention, comparators, outcomes, study design.

**Supplementary 2: Search Strategy**

## Database: PubMed <inception to August 31 2025>

***Search Strategy:***

| Search number | Query |
| --- | --- |
| 12 | ((((((((((((child[MeSH Terms]) OR (adolescent[MeSH Terms])) OR (children[Title/Abstract])) OR (pupils[Title/Abstract])) OR (student*[Title/Abstract])) OR (youth[Title/Abstract])) OR (junior[Title/Abstract])) OR (teenager[Title/Abstract])) OR (juvenile[Title/Abstract])) OR (pediatric[Title/Abstract])) AND (((((((((music therapy[MeSH Terms]) OR ("music therapy"[Title/Abstract])) OR (musicotherapy[Title/Abstract])) OR ("musical therapy"[Title/Abstract])) OR ("music treatment"[Title/Abstract])) OR ("musical-therapeutics"[Title/Abstract])) OR (music[Title/Abstract])) OR ("Sound therapy"[Title/Abstract])) OR ("Rhythm therapy"[Title/Abstract]))) AND ((depression[MeSH Terms]) OR (("depression"[Title/Abstract] OR "depressive"[Title/Abstract] OR "depressed"[Title/Abstract] OR "melancholia"[Title/Abstract] OR "dysphoria"[Title/Abstract] OR "despair"[Title/Abstract] OR "despondency"[Title/Abstract] OR "emotional depression"[Title/Abstract] OR "depressive symptom"[Title/Abstract])))) AND ((randomized controlled trial[pt] OR controlled clinical trial[pt] OR randomized[tiab] OR placebo[tiab] OR drug therapy[sh] OR randomly[tiab] OR trial[tiab] OR groups[tiab]) NOT (animals[mh] NOT humans[mh])) |
| 11 | (randomized controlled trial[pt] OR controlled clinical trial[pt] OR randomized[tiab] OR placebo[tiab] OR drug therapy[sh] OR randomly[tiab] OR trial[tiab] OR groups[tiab]) NOT (animals[mh] NOT humans[mh]) |
| 10 | (depression[MeSH Terms]) OR (("depression"[Title/Abstract] OR "depressive"[Title/Abstract] OR "depressed"[Title/Abstract] OR "melancholia"[Title/Abstract] OR "dysphoria"[Title/Abstract] OR "despair"[Title/Abstract] OR "despondency"[Title/Abstract] OR "emotional depression"[Title/Abstract] OR "depressive symptom"[Title/Abstract])) |
| 9 | ("depression"[Title/Abstract] OR "depressive"[Title/Abstract] OR "depressed"[Title/Abstract] OR "melancholia"[Title/Abstract] OR "dysphoria"[Title/Abstract] OR "despair"[Title/Abstract] OR "despondency"[Title/Abstract] OR "emotional depression"[Title/Abstract] OR "depressive symptom"[Title/Abstract])) |
| 8 | depression[MeSH Terms] |
| 7 | ((((((((music therapy[MeSH Terms]) OR ("music therapy"[Title/Abstract])) OR (musicotherapy[Title/Abstract])) OR ("musical therapy"[Title/Abstract])) OR ("music treatment"[Title/Abstract])) OR ("musical-therapeutics"[Title/Abstract])) OR (music[Title/Abstract])) OR ("Sound therapy"[Title/Abstract])) OR ("Rhythm therapy"[Title/Abstract]) |
| 6 | ("music therapy"[Title/Abstract])) OR (musicotherapy[Title/Abstract])) OR ("musical therapy"[Title/Abstract])) OR ("music treatment"[Title/Abstract])) OR ("musical-therapeutics"[Title/Abstract])) OR (music[Title/Abstract])) OR ("Sound therapy"[Title/Abstract])) OR ("Rhythm therapy"[Title/Abstract]) |
| 5 | music therapy[MeSH Terms] |
| 4 | (((((((((child[MeSH Terms]) OR (adolescent[MeSH Terms])) OR (children[Title/Abstract])) OR (pupils[Title/Abstract])) OR (student*[Title/Abstract])) OR (youth[Title/Abstract])) OR (junior[Title/Abstract])) OR (teenager[Title/Abstract])) OR (juvenile[Title/Abstract])) OR (pediatric[Title/Abstract]) |
| 3 | (children[Title/Abstract])) OR (pupils[Title/Abstract])) OR (student*[Title/Abstract])) OR (youth[Title/Abstract])) OR (junior[Title/Abstract])) OR (teenager[Title/Abstract])) OR (juvenile[Title/Abstract])) OR (pediatric[Title/Abstract]) |
| 2 | adolescent[MeSH Terms] |
| 1 | child[MeSH Terms] |

# Supplementary 3: Characteristics of studies and subjects included in the review

| **Study** | **Country/Region** | **Study design** | **Subjects  (intervention/ control)** | **Sex (boy/girl) (intervention/ control)** | **Mean age  (intervention/ control)** | **Baseline depression** | **Intervention detail** | | **Session duration** | **Training frequency** | **Duration** | **Adhenrence** | **Outcomes** |
| --- | --- | --- | --- | --- | --- | --- | --- | --- | --- | --- | --- | --- | --- |
|  |  |  |  |  |  |  | **Intervention group** | **Control group** |  |  |  |  |  |
| Cheung et al. (2019) | China (Hong Kong) | Open-label RCT | 60 (30/30) | 17/13 vs. 18/12 | 12.53±3.18 vs. 13.97±3.26 | 20.60±7.44 vs. 20.43±7.23 (CES-DC) | One-on-one instrumental music training (selecting instruments based on interests and abilities; step-by-step training of rhythm and musical skills, with lessons at home) | Unstructured leisure activities (such as playing cards or chess or watching online videos, TV series, or movies) | 45 min | 1 time/week | 52 weeks | 80%（24/30） | Depressive Symptoms, Self-Esteem, QoL |
| Currie et al. (2012) | Australia | Open-label RCT | 54 (28/26) | 28/0 vs. 26/0 | 13.8±0.89 | 16.4 ± 11.5 vs. 14.5 ± 8.3 (BDI) | Group psychotherapy with percussion as the core | Waiting list, no intervention | 50-60 min | 2 times/week | 10 weeks | ≥80% | Depressive Symptoms, Self-Esteem |
| Goldbeck et al. (2012) | Germany | Open-label RCT | 36 (18/18) | 10/8 vs. 9/9 | 10.2±1.3 vs. 10.4±1.2 | 10.8 ± 5.6 vs. 11.2 ± 5.8 (BDI) | Multimodal Music Therapy (MMT), including 3 individual music therapy sessions (60 minutes each) and 9 group music therapy sessions (90 minutes each) | Treatment as usual (TAU), which may include regular psychological counseling or follow-up | 60-90 min | 1 time/week | 12-15 weeks | 88.9%（16/18） | Depressive Symptoms, Anxiety Symptoms, QoL |
| Kwok et al. (2018) | China (Hong Kong) | Single-blind RCT | 106 (53/53) | 34/19 vs 40/13 | 13.63 ± 0.85 vs 13.53 ± 1.06 | NA | Resource-based music therapy integrating positive psychology (group format; including instrumental/percussion improvisation) | No intervention control (participating in daily extracurricular activities at school) | 90 min | 1 time/week | 8 weeks | 100% (53/53) | Anxiety Symptoms |
| Park et al. (2023) | South Korea | Open-label RCT | 36 (18/18) | 13/5 vs 10/8 | 11.97 ± 2.34 vs 12.14 ± 2.80 | 31.35 ± 9.27 vs 32.70 ± 10.21 (CDI) | Music therapy, including active music therapy and receptive music therapy | Standard care | 50 min | 2 times/week | 12 weeks | 60% (18/30) | Depressive Symptoms |
| Chen et al. (2019) | China (Taiwan) | Open-label RCT | 65 (32/33) | 3/29 vs. 6/27 | 12.19±0.40 vs. 12.24±0.44 | 13.34 ± 8.17 vs. 11.00 ± 11.09 (BDI-II) | Preference music therapy (create a top 50 playlist based on student preferences; listen to pop/classical music → choral karaoke (approximately 20 minutes) → calm classical music relaxation (medium tempo, approximately 5 minutes) | Typical routine, no specific intervention | 40 min | 2 times/week | 10 weeks | 96.7% (32/33) | Depressive Symptoms |
| Hilliard (2001) | USA | Open-label RCT | 18 (9/9) | NA | 8-12 | 12.44 ± 3.18 vs 13.33 ± 5.16 (DSRS) | Group music therapy, focusing on rhythm/percussion and rap | No-contact blank control | 60 min | 1 time/week | 8 weeks | 100% (9/9) | Depressive Symptoms |
| Cheung et al. (2024) | China (Hong Kong) | Single-blind RCT | 64 (32/32) | 20/12 vs 17/15 | 9.6 ± 1.6 vs 9.4 ± 1.3 | 22.5 ± 8.9 vs 23.3 ± 8.4 (CES-DC) | Instrumental group training (professional musicians teach, keyboard/ukulele/guitar assigned according to interest/ability) | No intervention during the study | 60 min | 1 time/week | 24 weeks | 62/64（96.9%） | Depressive Symptoms, Self-Esteem, QoL |
| Porter et al. (2016) | Northern Ireland, UK | Single-blind RCT | 251 (123/128) | 55/68 vs 56/72 | 12.7 ± 2.5 vs 12.7 ± 2.4 | 23.6 ± 12.4 vs 25.4 ± 14.1 (CES-DC) | Music therapy (Free Improvisation), individual interview; participants choose an instrument to express their feelings that day and interact with the therapist | Usual care: psychiatric consultation and/or medication | 30 min | 1 time/week | 12 weeks | 38% (47/123) | Depressive Symptoms, Self-Esteem |

RCT: Randomized Controlled Trial, CES-DC: Children's Depression Scale – Chinese version, BDI: Beck Depression Inventory, MMT: Multimodal Music Therapy, TAU: Treatment as Usual, CDI: Children's Depression Inventory, NA: Not Available, QoL: Quality of Life, DSRS: Depressive Symptom Rating Scale.

# Supplementary 4: Risk of Bias

## Table 4.1 The risk of bias assessment for the individual included studies

| **Study** | Randomization process | Deviations from intended interventions | Missing outcome data | Measurement of the outcome | Selection of the reported result | Overall Bias |
| --- | --- | --- | --- | --- | --- | --- |
| Cheung et al. (2019) | Some concerns | Some concerns | Some concerns | Low | Low | Some concerns |
| Currie et al. (2012) | Low | Some concerns | Low | Low | Low | Some concerns |
| Goldbeck et al. (2012) | Low | Low | Low | Low | Low | Low |
| Kwok et al. (2018) | Low | Low | Low | Low | Low | Low |
| Park et al. (2023) | Low | Low | Low | Low | Low | Low |
| Chen et al. (2019) | Low | Some concerns | Some concerns | Low | Low | Some concerns |
| Hilliard (2001) | Low | Low | Low | Low | Low | Low |
| Cheung et al. (2024) | Low | Some concerns | Low | Low | Low | Some concerns |
| Porter et al. (2016) | Low | Low | Low | Low | Low | Low |

# Supplementary 5: Publication bias

Figure 5.1 The funnel plot of depression. The result of Egger test showed the p=0.109.

Figure 5.2 The funnel plot of anxiety.

Figure 5.3 The funnel plot of self-esteem. The result of Egger test showed the p=0.129.

Figure 5.4 The funnel plot of QoL The result of Egger test showed the p=0.824.

# Supplementary 6: Sensitive analysis

Figure 5.1 The sensitive analysis of depression.

Figure 5.2 The sensitive analysis of anxiety.

Figure 5.3 The sensitive analysis of self-esteem.

Figure 5.4 The sensitive analysis of QoL.

**Supplementary Table 5.1. Sensitivity analysis excluding studies with “some concerns” risk of bias.**

| **Outcome** | **Main analysis, all studies** | **Trials excluded due to “some concerns”** | **Low-risk trials retained** | **Sensitivity estimate after exclusion** |
| --- | --- | --- | --- | --- |
| Depressive symptoms | 8 studies; n = 584; SMD = −0.55, 95% CI −0.71 to −0.38; I² = 25.4% | 4 studies | 4 studies; n = 341 | SMD = −0.39, 95% CI −0.62 to −0.15; I² = 0% |
| Anxiety symptoms | 2 studies; n = 142; SMD = −0.42, 95% CI −0.75 to −0.08; I² = 0% | 0 studies | 2 studies; n = 142 | SMD = −0.42, 95% CI −0.75 to −0.08; I² = 0% |
| Self-esteem | 4 studies; n = 429; SMD = 0.45, 95% CI 0.26 to 0.64; I² = 61.0% | 3 studies | 1 study; n = 251 | SMD = 0.26, 95% CI 0.01 to 0.50 |
| Quality of life | 3 studies; n = 160; SMD = 0.69, 95% CI 0.37 to 1.01; I² = 5.2% | 2 studies | 1 study; n = 36 | SMD = 0.46, 95% CI −0.19 to 1.10 |
